# Synthesis of Zwitter-Ionic Conjugate of *Nido*-Carborane with Cholesterol

**DOI:** 10.3390/molecules26216687

**Published:** 2021-11-05

**Authors:** Anna A. Druzina, Olga B. Zhidkova, Nadezhda V. Dudarova, Natalia A. Nekrasova, Kyrill Yu. Suponitsky, Sergey V. Timofeev, Vladimir I. Bregadze

**Affiliations:** 1 A.N. Nesmeyanov Institute of Organoelement Compounds, Russian Academy of Sciences, 28 Vavilov Str., 119991 Moscow, Russia; Zolga57@mail.ru (O.B.Z.); nadezjdino_96@mail.ru (N.V.D.); neksova_na@list.ru (N.A.N.); kirshik@yahoo.com (K.Y.S.); timofeev@ineos.ac.ru (S.V.T.); bre@ineos.ac.ru (V.I.B.); 2 M.V. Lomonosov Institute of Fine Chemical Technology, MIREA—Russian Technological University, 86 Vernadsky Av., 119571 Moscow, Russia; 3 Basic Department of Chemistry of Innovative Materials and Technologies, G.V. Plekhanov Russian University of Economics, 36 Stremyannyi Line, 117997 Moscow, Russia

**Keywords:** *nido*-carborane, “click” reaction, cholesterol, X-ray diffraction

## Abstract

9-HC≡CCH_2_Me_2_N-*nido*-7,8-C_2_B_9_H_11_, a previously described carboranyl terminal alkyne, was used for the copper(I)-catalyzed azide-alkyne cycloaddition with azido-3β-cholesterol to form a novel zwitter-ionic conjugate of *nido*-carborane with cholesterol, bearing a 1,2,3-triazol fragment. The conjugate of *nido*-carborane with cholesterol, containing a charge-compensated group in the linker, can be used as a precursor for the preparation of liposomes for BNCT (Boron Neutron Capture Therapy). The solid-state molecular structure of a *nido*-carborane derivative with the 9-Me_2_N(CH_2_)_2_Me_2_N-*nido*-7,8-C_2_B_9_H_11_ terminal dimethylamino group was determined by single-crystal X-ray diffraction.

## 1. Introduction

Due to their unique structure and chemical properties, as well their wide range of applications, polyhedral boron hydrides attract the continued interest of researchers working in various fields [1,2,3]. *Nido*-Carborane or 7,8-dicarba-*nido*-undecaborate anion and its derivatives have highly polarizable spherical aromaticity as a result of their σ-delocalized electron density [4,5]. Therefore, they display characteristic electronic properties [6], and thermal [7], chemical and photochemical stability [8]. All these features make them interesting systems for use in such fields as materials science [9,10] and medicinal chemistry [11,12,13,14]. Boron neutron capture therapy (BNCT) for cancer is one of the most significant applications of *nido*-carborane derivatives in medicine [15].

The basic concept of BNCT for cancer, proposed for the first time by Gordon L. Locher in 1936, is based on the selective accumulation of the non-radioactive isotope ^10^B in tumor cells, and their subsequent treatment with a flux of thermal neutrons [16]. On absorption of a thermal neutron by a ^10^B atom, an excited ^11^B atom is formed, which almost immediately undergoes a fission reaction, producing two high-energy heavy ions (^4^He^2+^ and ^7^Li^3+^) that selectively destroy tumor cells, while not causing serious damage to the surrounding normal cells [17]. To ensure the successful development of BNCT, the selective delivery and high accumulation of boron in the tumor tissue, at a therapeutic concentration (20–35 µg ^10^B/g), are required for subsequent irradiation with thermal neutrons [17]. For this purpose, the development of efficient ^10^B delivery carriers to tumors is important in BNCT.

The promising trend towards the achievement of the necessary therapeutic concentrations of boron in the tumor is enabled by the use of *nido*-carborane derivatives containing high contents of boron atoms in their molecules [15,18,19]. Another promising trend is the development of various nanomaterials, such as liposomes, that could be used as both boron host molecules and for the targeted delivery of boron to cancer cells [20,21].

Liposomes are artificially constructed spherical vesicles consisting of a phospholipid bilayer [22]. First discovered in 1961 by Alec Bangham, liposomes are now being studied for their ability to overcome cell membranes and transport boron clusters into a cancerous tumor. There are examples of the production of liposomes based on polyhedral boron hydrides containing borane and carborane derivatives both in the aqueous core and in the composition of the lipid bilayer [23,24,25]. The main difference between tumor and normal cells is the presence of a phospholipid/cholesterol shell with a diameter of approximately 15–20 nm, which is filled with cholesterol and glyceryl esters of long-chain alkyl carboxylic acids. This difference is based on an increase in the cholesterol requirement of tumor cells to promote the formation of new membranes. Therefore, the design of stable biocompatible boron-containing cholesterol nanostructures for the further creation of liposomal agents that contain derivatives of polyhedral boron hydrides is an effective approach that can solve the problem of the selective delivery of boron into tumor cells required to carry out BNCT. It was also recently shown that the inclusion of lipophilic boron-containing species in the bilayer of liposomes provides an attractive means of increasing the total boron content in liposomes contained within the formulation [26,27]. In addition, it was found that the encapsulation of *nido*-carborane with PEGylated liposome via the hydration of thin lipid films significantly suppresses tumors in BNCT [28].

It should be noted that the penetration of boronated liposomes through biological membranes, and their accumulation and retention in cells, largely depend on their charge. It is known that positively charged liposomes have better penetration through biological membranes than negatively charged ones. Positively charged liposomes containing carborane derivatives were found to be the most efficient delivery system for rat colon carcinoma and murine melanoma cell lines, as compared with negatively charged liposomes [29,30,31]. The high accumulation of such liposomes was probably due to favorable electrostatic interactions with the negatively charged outer leaflets of mammalian plasma membranes. This inspired us to synthesize positively charged *nido*-carborane derivatives with cholesterol for further use in the form of liposomes. The design of such compounds is based on the introduction of two ammonium centers, the first of which compensates for the negative charge of the *nido*-carborane cluster, and the second one provides the overall positive charge of the molecule.

There are only a few examples of lipids with polyhedral boron hydrides that contain molecules with zwitter-ionic characteristics [32,33]. Here, we use the “click” methodology to approach the synthesis of a novel zwitter-ionic *nido*-carboranyl-cholesterol conjugate. The hydrophilic part of such lipids contains a *nido*-carborane cluster, while the lipophilic part contains cholesterol. The resulting conjugate can be used to produce boron-containing liposomes as potential drugs for BNCT.

## 2. Results and Discussion

### 2.1. Synthesis of Nido-Carboranyl Cholesterol Bearing a 1,2,3-Triazole Fragment

The synthesis of biologically active molecules is a very important area of bioorganic chemistry. Among the methods for obtaining bioconjugates, the Cu(I)-catalyzed 1,3-dipolar [3+2] cycloaddition reaction of alkynes to azides is widely used, leading to the formation of 1,2,3 triazoles; this is known as the “click”-reaction” [34,35,36]. Earlier, the “click”-reaction was successfully used to obtain a wide range of conjugates of polyhedral boron hydrides with various biologically active molecules, such as nucleosides [37] and chlorine *e*_6_ [38], as well as derivatives of cholesterol based on cobalt/iron bis(dicarbollide) [26,27,33,39], *closo*-dodecaborate dianion [40] and *nido*-carborane [41]. In this work, we used cholesterol derivatives, as well as 3β-(2-azido-ethoxy)cholest-5-ene and *nido*-carboranyl derivatives, that contained different spacers between the boron cage and the terminal acetylene group.

**Figure 1:**
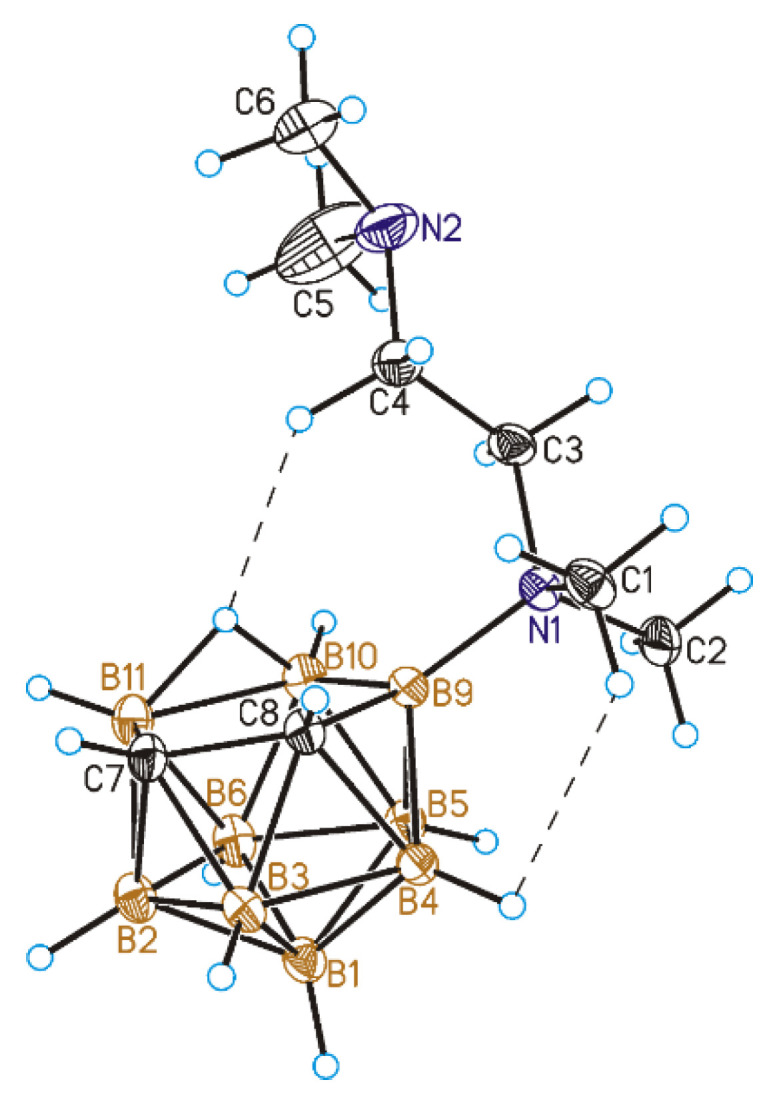
Molecular view of 9-Me_2_N(CH_2_)_2_Me_2_N-*nido*-7,8-C_2_B_9_H_11_ **4** presented by thermal ellipsoids at 50% probability level. Shortened H4A…H12 and H4…H1A contacts are shown by dashed lines.

Thus, to obtain a target positively charged conjugate by means of the “click” reaction, a previously described terminal alkyne based on *nido*-carborane derivative, with two ammonium centers in its spacer [9-HC≡CCH_2_Me_2_N(CH_2_)_2_Me_2_N-*nido*-7,8-C_2_B_9_H_11_]Br (**1**) [42], was used. However, it was found that the reaction of acetylene **1** with azido-3β-cholesterol **2** in the refluxing of ethanol in the presence of diisopropylethylamine (DIPEA) and a catalytic amount of CuI did not lead to the desired conjugate **3**. During this reaction, we observed the elimination of the propargyl fragment of compound **1** and the formation of the *nido*-carborane derivative with the terminal dimethylamino group 9-Me_2_N(CH_2_)_2_Me_2_N-*nido*-7,8-C_2_B_9_H_11_ (**4**). This can be explained by the acetylene-allen rearrangement under the action of DIPEA. The structure of compound **4** was confirmed by ^1^H, ^11^B and ^13^C NMR spectra. The spectral characteristics of **4** are in good agreement with the data given in the literature [42]. The structure of 9-Me_2_N(CH_2_)_2_Me_2_N-*nido*-7,8-C_2_B_9_H_11_ **4** was additionally confirmed via single-crystal X-ray diffraction study (Figure 1).

**Scheme 1:**
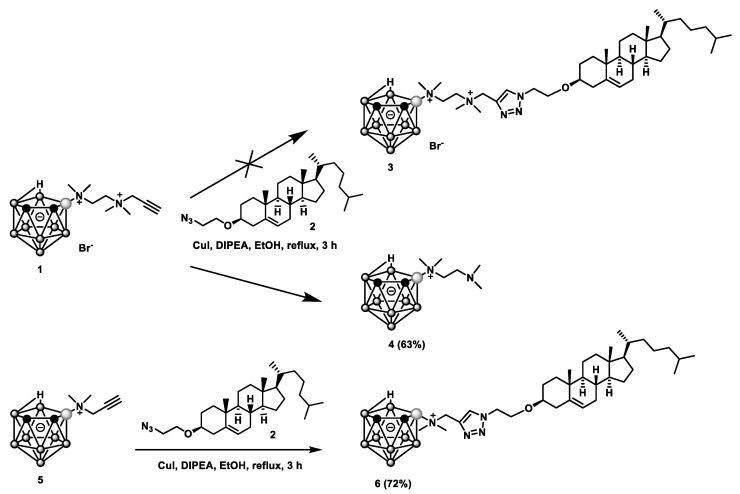
Synthesis of zwitter-ionic *nido*-carboranyl conjugate with cholesterol **6**.

Further, we decided to use a more stable terminal *nido*-carboranyl alkyne with one ammonium center, 9-HC≡CCH_2_Me_2_N-*nido*-7,8-C_2_B_9_H_11_ (**5**) [42]. The usage of this alkyne excluded the elimination of the propargyl fragment in compound **5**. Indeed, the reaction of acetylene **5** with azido-3β-cholesterol **2**, under the same conditions as for compound **1**, produced the novel conjugate of *nido*-carboranyl cholesterol **6** with a zwitter-ionic character of its target molecule (Scheme 1).

The structure of the obtained conjugate **6** was confirmed by ^1^H, ^11^B, ^13^C NMR, IR and high-resolution mass-spectrometry. The ^1^H and ^13^C NMR spectra of compound **6**, along with the signals for the heteroaliphatic chain, contained signals that were characteristic of the triazole ring. In the ^1^H NMR spectrum, the signals for the protons of the C*H* group of triazole appeared at 8.37 ppm for conjugate **6**. For 1,2,3-triazole **6**, the ^13^C NMR spectrum showed signals for two carbon atoms of the triazole fragment at 140.6 ppm (the “nodal” atom) and at 123.3 ppm. In the ^1^H NMR spectrum, the signal of the methylene group next to the triazole cycle was observed at 4.63 ppm and the characteristic signals of the *Me*_2_N hydrogens appeared at 3.02 and 3.08 ppm. The characteristic signal for the proton at the double bond of the steroid core (C*H*st) of the conjugate was observed in the region of 5.33 ppm. The ^11^B NMR spectrum of **6** contained a pattern of eight signals (one singlet at 5.8 ppm and seven doublets at −5.7, −17.4, −19.4, −24.7, −26.9, −38.2 and −38.8 ppm), which demonstrates the absence of a plane of symmetry and unambiguously confirms the *nido* form. In the ^1^H-NMR spectra, the signal of the *extra*-hydrogen, as expected, was observed at approx. −3.2 ppm. In addition, the signals of the C*H*_carb_ groups in the ^1^H NMR spectra of **6** appeared as broad singlets at 2.81 and 2.06 ppm; in the ^13^C NMR spectra, the signals of *C*H_carb_ groups appeared at 33.6 and 39.7 ppm. The IR spectrum of compound **5** exhibited absorption band characteristic of the BH group at 2549 cm^−1^ and of the triazole ring at 1421 cm^−1^.

Based on synthesized zwitter-ionic compound, the preparation of the boronated liposomes was planned in order to deliver boron clusters into a cancer cell for the BNCT experiment.

### 2.2. Single-Crystal X-ray Diffraction Studies

The molecular and crystal structure of charge-compensating *nido*-carborane derivative with terminal dimethylamino group 9-Me_2_N(CH_2_)_2_Me_2_N-*nido*-7,8-C_2_B_9_H_11_ (**4**) was determined by means of a single-crystal X-ray diffraction study (Figure 1). Crystals of **4** that were suitable for single-crystal X-ray analysis were obtained from the CDCl_3_ solution in the NMR tube.

**Figure 2:**
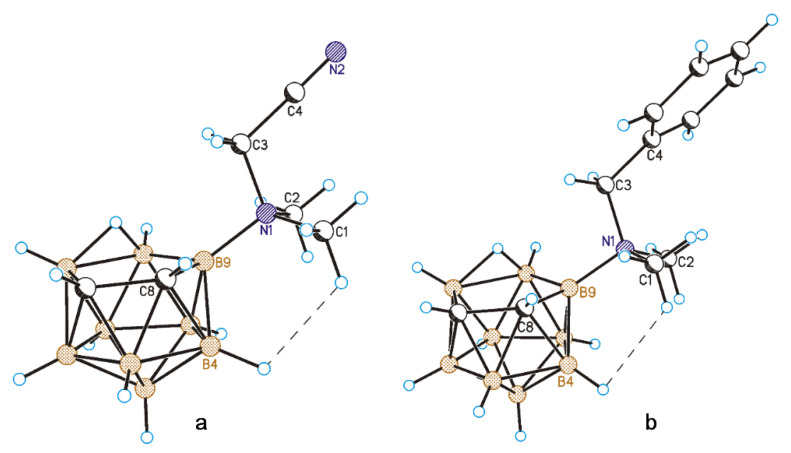
Molecular view of 9-NC≡CCH_2_Me_2_N-*nido*-7,8-C_2_B_9_H_11_ (**a**) and 9-PhCH_2_Me_2_N-*nido*-7,8-C_2_B_9_H_11_ (**b**). Only necessary numbering is provided. Shortened H4…H1A contacts are shown by dashed lines.

The side substituent was relatively flexible and had the potential to form shortened C-H…H-B contacts with carborane cages, which would have influenced its orientation relative to the cage. On the other hand, relative orientation could have been affected by the crystal packing. Torsion angles and shortened H…H contacts, which defined the relative orientation, are provided in Table 1. In Table 1, we also included two recently studied compounds (Figure 2) [42], with similar substituents, at the same position in the carborane cage, as well as the calculated molecular geometry results obtained in an isolated state for all three compounds. Calculations were carried out in terms of density functional theory using the PBE0 function, which is widely used for the geometric optimization of a variety of classes of compounds [43,44,45,46].

Crystal packing analysis of the recently studied 9-NC≡CCH_2_Me_2_N-*nido*-7,8-C_2_B_9_H_11_ and 9-PhCH_2_Me_2_N-*nido*-7,8-C_2_B_9_H_11_ [42] revealed that the crystal packing of 9-PhCH_2_Me_2_N-*nido*-7,8-C_2_B_9_H_11_ is mostly stabilized by numerous weak van-der-Waals intermolecular interactions, while, in 9-NC≡CCH_2_Me_2_N-*nido*-7,8-C_2_B_9_H_11_, relatively strong π…π interactions between cyano groups were observed. In the case of 9-Me_2_N(CH_2_)_2_Me_2_N-*nido*-7,8-C_2_B_9_H_11_ **4**, we found that the side substituent formed one slightly shortened C-H…H-B contact (2.31Å) while all the other intermolecular interactions were of the van-der-Waals type. Those observations agree well with the results shown in Table 1.

It can be seen that, in all three compounds, the crystal packing influenced the molecular structure. This influence was relatively small (at least, all shortened H…H contacts were preserved upon the transferring of a molecule from an isolated state to a crystal) and appeared to be more pronounced for 9-NC≡CCH_2_Me_2_N-*nido*-7,8-C_2_B_9_H_11_, for which π…π intermolecular interactions were observed.

## 3. Materials and Methods

### 3.1. General Methods

[9-HC≡CCH_2_Me_2_N(CH_2_)_2_Me_2_N-*nido*-7,8-C_2_B_9_H_11_]Br **1 [42]**, azido-3β-cholesterol **2** [39,47] and 9-HC≡CCH_2_Me_2_N-*nido*-7,8-C_2_B_9_H_11_ **5 [42]** were prepared according to the literature. Cholesterol (Fisher Scientific, Loughborough, UK), diisopropylethylamine (Carl Roth GmbH, Karlsruhe, Germany) and CuI (PANREAC QUIMICA SA, Barcelona, Spain) were used without further purification. Ethanol, CH_3_CN and CH_2_Cl_2_ were commercial reagents of analytical grade. The reaction progress was monitored by thin-layer chromatography (Merck F245 silica gel on aluminum plates) and visualized using 0.1% PdCl_2_ in 3 M HCl. Acros Organics silica gel (0.060–0.200 mm) was used for column chromatography. The NMR spectra at 400.1 MHz (^1^H), 128.4 MHz (^11^B) and 100.0 MHz (^13^C) were recorded with a Bruker Avance-400 spectrometer (Bruker, Karlsruhe-Zurich, Switzerland-Germany). The residual signal of the NMR solvent relative to Me_4_Si was taken as the internal reference for the ^1^H- and ^13^C-NMR spectra. ^11^B-NMR spectra were referenced using BF_3_*Et_2_O as external standard. Infrared spectra were recorded on an IR Prestige-21 (SHIMADZU) instrument. High resolution mass spectra (HRMS) were measured on a micrOTOF II (Bruker Daltonic, Bremen, Germany) instrument using electrospray ionization (ESI). The measurements were conducted in a positive ion mode (interface capillary voltage −4500 V), with a mass range from *m*/*z* 50 to *m*/*z* 3000; external or internal calibration was performed with the ESI Tuning Mix, produced by Agilent. A syringe injection was used for the addition of the solutions to acetonitrile (flow rate 3 µL/min). Nitrogen was applied as a dry gas; the interface temperature was set at 180 °C.

The X-ray experiment for **4** was carried out using the SMART APEX2 CCD diffractometer (λ(Mo-Kα) = 0.71073 Å, graphite monochromator, *ω*-scans) at 120K. The collected data were processed using the SAINT and SADABS programs that were incorporated into the APEX2 program package [48]. The structure was solved using direct methods and was refined by the full-matrix least-squares procedure against *F*^2^ via an anisotropic approximation. The refinement was carried out with the SHELXTL program [49]. The CCDC number 2113014 contains the supplementary crystallographic data (Supplementary Materials) for this paper. These data can be obtained free of charge via www.ccdc.cam.ac.uk/data_request/cif (accessed on 14 July 2021). 

### 3.2. General Procedure for the Synthesis of the Compounds ***4*** and ***6***

A mixture of azido-3β-cholesterol **2**, alkyne, diisopropylethylamine and CuI in ethanol was heated under reflux for 3 h. Then, the reaction mixture was cooled to room temperature and was passed through ca. 2–3 cm of silica on a Schott filter. Then, the solvent was removed in vacuo. The crude product was purified on a silica column using CH_2_Cl_2_-CH_3_CN as an eluent to provide the desired products **4** or **6**.

### 3.3. Synthesis of 9-Me_2_N(CH_2_)_2_Me_2_N-Nido-7,8-C_2_B_9_H_11_ (***4***)

9-Me_2_N(CH_2_)_2_Me_2_N-*nido*-7,8-C_2_B_9_H_11_ was prepared from azido-3β-cholesterol **2** (0.21 g, 0.45 mmol), boronated alkyne **1** (0.20 g, 0.45 mmol), diisopropylethylamine (1 mL, 0.74 g, 5.73 mmol) and CuI (0.009 g, 0.05 mmol) in 15 mL of ethanol. The product was purified on a silica column using CH_2_Cl_2_-CH_3_CN (1:1) as an eluent to give the white solid of **4** (0.07 g, yield 63%). The NMR data of **4** are in good agreement with the data in the literature [39]. *Crystallographic data*: crystals of **4**. C_8_H_27_B_9_N_2_ are monoclinic, space group *C*2*c*: *a* = 32.1867(12)Å, *b* = 6.6299(3)Å, *c* = 16.1995(6)Å, *β* = 116.1480(10)°, *V* = 3103.1(2) Å^3^, *Z* = 8, *M* = 248.60, *d*_cryst_ = 1.064 g⋅cm^−3^. *wR*2 = 0.1369 calculated on *F*^2^*_hkl_* for all 4947 independent reflections with 2θ < 62.0°, (*GOF* = 1.117, *R* = 0.0546 calculated on *F_hkl_* for 3942 reflections with *I* > 2*σ*(*I*)).

### 3.4. Synthesis of 9-3β-Chol-O(CH_2_)_2_N_3_-CH-C-(CH_2_)Me_2_N-Nido-7,8-C_2_B_9_H_11_ (***6***)

9-3β-Chol-O(CH_2_)_2_N_3_-CH-C-(CH_2_)Me_2_N-*nido*-7,8-C_2_B_9_H_11_ was prepared from azido-3β-cholesterol **2** (0.09 g, 0.21 mmol), boronated alkyne **5** (0.06 g, 0.21 mmol), diisopropylethylamine (0.5 mL, 0.40 g, 2.87 mmol) and CuI (0.004 g, 0.02 mmol) in 7 mL of ethanol. The product was purified on a silica column using CH_2_Cl_2_-CH_3_CN (7:1) as an eluent to give the white solid of **6** (0.10 g, yield 72%). ^1^H NMR (400 MHz, acetone-*d*_6_) δ 8.37 (1H, s, CC*H*N_3_), 5.34 (1H, m, C_st_(6)H), 4.69 (4H, s, -OC*H_2_*-C*H_2_*N_3_), 3.96 (2H, s, C*H_2_*NMe_2_), 3.17 (1H, m, C_st_(3)H), 3.08 (3H, s, N*Me*_2_), 3.02 (3H, s, N*Me*_2_), 2.81 (1H, br. s, C*H_carb_*), 2.31 (1H, m, C_st_(H)), 2.40 (1H, m, C_st_(H)), 2.06 (1H, br. s, C*H_carb_*), 1.86 (6H, m, C_st_(H),), 1.57–1.03 (22H, m, C_st_(H)), 1.00 (3H, s, C_st_(19)H_3_), 0.96 (3H, d, *J* = 6.3 Hz, C_st_(21)H_3_,), 0.89 (3H, s, C_st_(26)H_3_), 0.88 (3H, s, C_st_(27)H_3_), 0.73 (3H, s, C_st_(18)H_3_), −3.16 (1H, br. s., *H_extra_*) ppm; ^11^B (128 MHz, acetone-*d*_6_) δ 5.8 (1B, s), −5.7 (1B, d, *J* = 148), −17.4 (2B, unsolved d), −19.4 (1B, unsolved d), −24.7 (1B, d, *J* = 142), −26.9 (1B, d, *J* = 147), −32.2 (1B, d, *J* = 116), −38.8 (1B, d, *J* = 144) ppm; ^13^C NMR (101 MHz, acetone-*d*_6_) δ 140.5 (*C*CHN_3_), 137.3 (C_st_(5)), 123.3 (C*CH*N_3_), 121.5 (C_st_(6)), 79.1 (C_st_(3)), 66.0 (CCHN_3_-*CH_2_*), 61.2 (O-*CH_2_*), 56.7 (C_st_(14)), 56.1 (C_st_(17)), 51.9 (N*Me*_2_), 50.9 (N*Me*_2_), 50.7 (C_st_(9)), 50.3 (*C*H_2_NMe_2_), 42.2 (C_st_(4)), 39.7 (C_carb_), 39.4 (C_st_(12)), 38.9 (C_st_(13)), 37.0 (C_st_(24)), 36.6 (C_st_(1)), 36.1 (C_st_(10)), 35.7 (C_st_(22)), 33.6 (C_carb_), 31.8 (C_st_(20)), 31.7 (C_st_(8)), 28.2 (C_st_(2)), 28.0 (C_st_(7)), 27.8 (C_st_(16)), 24.0 (C_st_(25)), 23.6 (C_st_(15)), 22.2 (C_st_(23)), 22.0 (C_st_(26), C_st_(27)), 20.9 (C_st_(11)), 18.8 (C_st_(19)), 18.2 (C_st_(21)), 11.3 (C_st_(18)) ppm. IR (solid): ν = 2549 cm^−1^ (BH), 1421 cm^−1^ (triazole), HRMS-ESI^+^*m/z* for [C_34_H_64_B_9_N_4_O + H]^+^ calcd 642.6016, found: 642.6028.

## 4. Conclusions

In this work, the “click” reaction of 9-HC≡CCH_2_Me_2_N-*nido*-7,8-C_2_B_9_H_11_ with azido-3β-cholesterol, in a good yield, was conducted to prepare a novel zwitter-ionic conjugate of *nido*-carborane with cholesterol. We also studied the behavior of the *nido*-carborane derivative [9-HC≡CCH_2_Me_2_N(CH_2_)_2_Me_2_N-*nido*-7,8-C_2_B_9_H_11_]Br in the copper(I)-catalyzed azide-alkyne cycloaddition reaction with azido-3β-cholesterol, and revealed that during this process, in the presence of a strong base, acetylene-allen rearrangement occurred, resulting in the elimination of the propargyl fragment in carbonyl acetylene and the formation of 9-Me_2_N(CH_2_)_2_Me_2_N-*nido*-7,8-C_2_B_9_H_11_. The solid-state molecular structure of this previously described compound was determined by means of single-crystal X-ray diffraction studies. Comparing the structure of 9-Me_2_N(CH_2_)_2_Me_2_N-*nido*-7,8-C_2_B_9_H_11_ with the previously described analogs shows that the flexibility of side substituent does not significantly affect the crystal packing. Based on synthesized zwitter-ionic conjugate, the preparation of the boronated liposomes was planned in order to deliver boron clusters into a cancer cell for BNCT experiments.

## Figures and Tables

**Table 1:** Torsion angles (degree) and shortened H…H contacts (Å) defining the orientation of side substituents relative to the carborane cage for the compounds 9-Me_2_N(CH_2_)_2_Me_2_N-*nido*-7,8-C_2_B_9_H_11_ **4**, 9-NC≡CCH_2_Me_2_N-*nido*-7,8-C_2_B_9_H_11_ and 9-PhCH_2_Me_2_N-*nido*-7,8-C_2_B_9_H_11_.

Torsion Angle or H…H Contact	9-Me_2_N(CH_2_)_2_Me_2_N-*Nido*-7,8-C_2_B_9_H_11_ 4		9-NC≡CCH_2_Me_2_N-*Nido*-7,8-C_2_B_9_H_11_		9-PhCH_2_Me_2_N-*Nido*-7,8-C_2_B_9_H_11_	
	X-ray	DFT	X-ray	DFT	X-ray	DFT
C8-B9-N1-C1	19.4(2)	28.3	50.4(2)	36.0	35.3(2)	41.9
C8-B9-N1-C2	138.4(2)	148.0	172.4(2)	157.8	155.8(2)	162.2
C8-B9-N1-C3	−103.6(2)	−94.7	−69.2(2)	−84.1	−84.8(2)	−78.8
B9-N1-C3-C4	64.0(2)	63.9	177.4(2)	179.6	171.9(2)	−179.7
N1-C3-C4-N2	178.3(2)	−170.6	–	–	–	–
C3-C4-N2-C5	74.1(2)	81.5	–	–	–	–
C3-C4-N2-C6	−163.1(2)	−152.8	–	–	–	–
H4…H1A	2.33	2.29	2.26	2.24	2.33	2.27
H4A…H12	2.38	2.39	–	–	–	–

## Data Availability

Not applicable.

## Supplementary Materials

The following are available online. Figures S1–S6 NMR, IR and high-resolution mass spectra of compound **6**.

## Sample Availability

Samples of compound **6** are available from the authors.
