# Supplementary material for: Synthesis of Zwitter-Ionic Conjugate of Nido-Carborane with Cholesterol
Source: Molecules. 2021 Nov 5;26(21):6687. doi: 10.3390/molecules26216687 (PMC8588508; doi:10.3390/molecules26216687)
Supplement: Supplementary file 1 [file molecules-26-06687-s001.zip › Supporting Information.pdf]

SUPPORTING INFORMATION

Synthesis of zwitter-ionic conjugate of *nido*-carborane with cholesterol

Anna A. Druzina[1,*], Olga B. Zhidkova[1], Nadezhda V. Dudarova[1], Natalia A. Nekrasova[1,2], Kyrill Yu. Suponitsky[1,3], Sergey V. Timofeev[1] and Vladimir I. Bregadze[1]

[1] A.N. Nesmeyanov Institute of Organoelement Compounds, Russian Academy of Sciences, 28 Vavilov Str., 119991 Moscow, Russia; Zolga57@mail.ru (O.B.Z.); nadezjdino_96@mail.ru (N.V.D.); neksova_na@list.ru(N.A.N.); kirshik@yahoo.com (K.Yu.S.); timofeev@ineos.ac.ru (S.V.T.); bre@ineos.ac.ru (V.I.B).

[2] M.V. Lomonosov Institute of Fine Chemical Technology, MIREA - Russian Technological University, 86 Vernadsky Av., 119571, Moscow, Russia; neksova_na@list.ru(N.A.N.)

[3] G.V. Plekhanov Russian University of Economics, 36 Stremyannyi Line, 117997, Moscow, Russia; kirshik@yahoo.com (K.Yu.S.)

* Correspondence: ilinova_anna@mail.ru; Tel.: +7-926-404-5566 (A.A.D.)

^{1}H, ^{11}B and ^{13}C NMR, IR and high-resolution mass spectra of compounds **6**

Display Report

Analysis Info

Analysis Name	D:\Data\Chizhov\INEOS\Laskova\da-041_&clb.d	Acquisition Date	29.09.2020 15:27:17
Method	tune_wide.m	Operator	BDAL@DE
Sample Name	/CHIZ DA-041	Instrument	maXis 43
Comment	CH3CN !00 %, dil. 200, calibrant added		

Acquisition Parameter

Source Type	ESI	Ion Polarity	Positive	Set Nebulizer	0.5 Bar
Focus	Active	Set Capillary	4500 V	Set Dry Heater	180 °C
Scan Begin	50 m/z	Set End Plate Offset	-500 V	Set Dry Gas	4.0 l/min
Scan End	3000 m/z	Set Collision Cell RF	1200.0 Vpp	Set Divert Valve	Waste

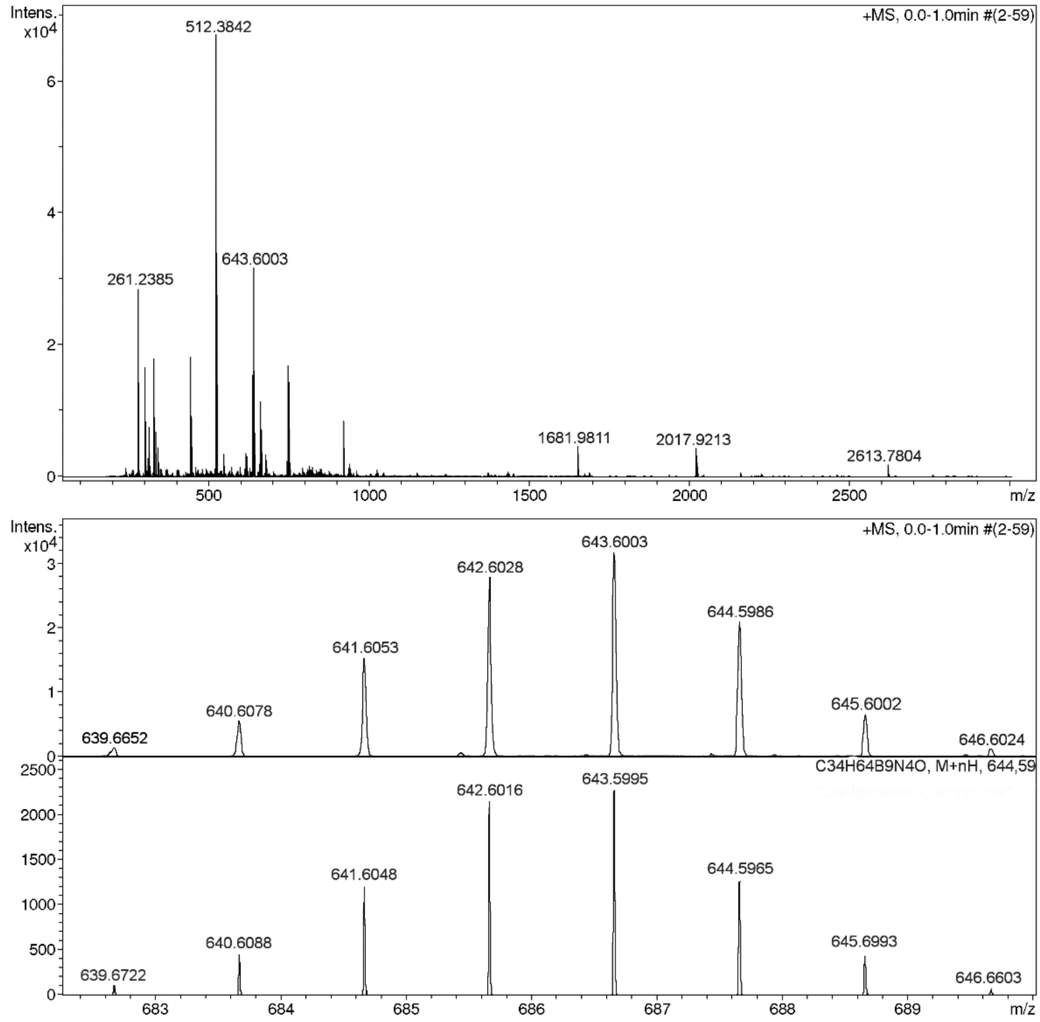

Figure S1. ESI-HRMS spectrum of compound **6**

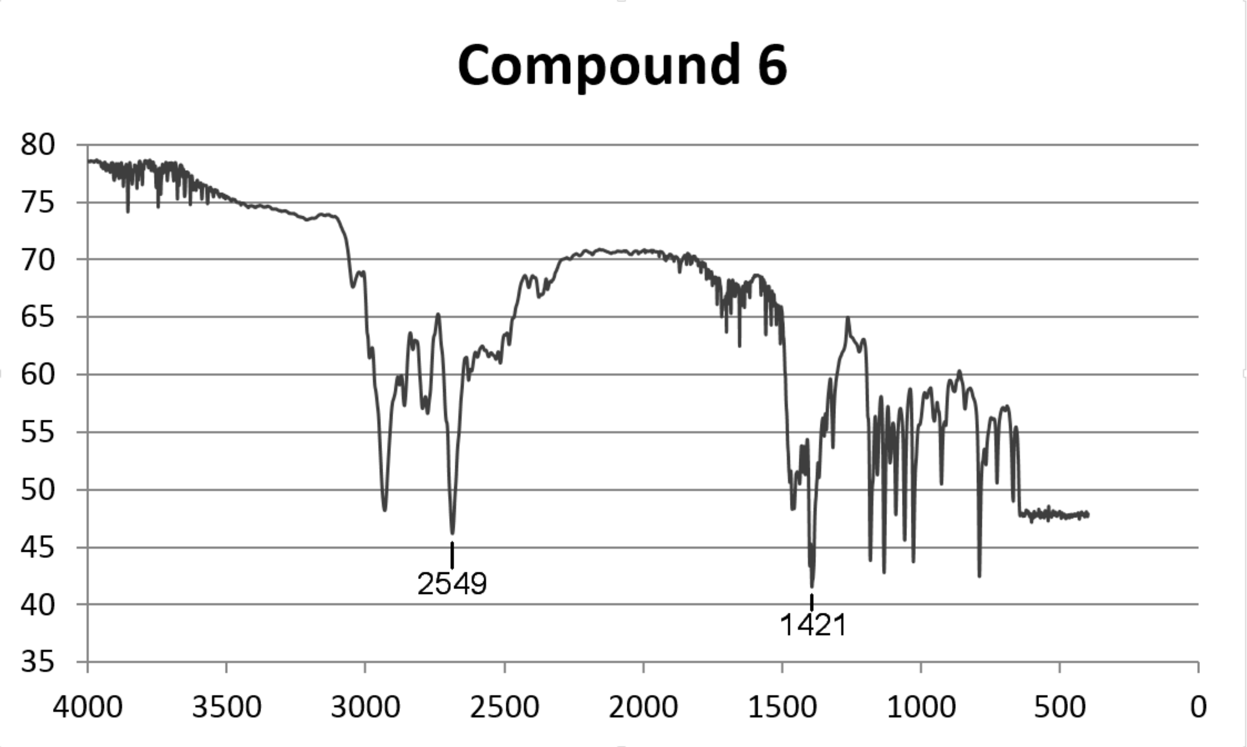

Figure S2. IR spectrum of compound **6**

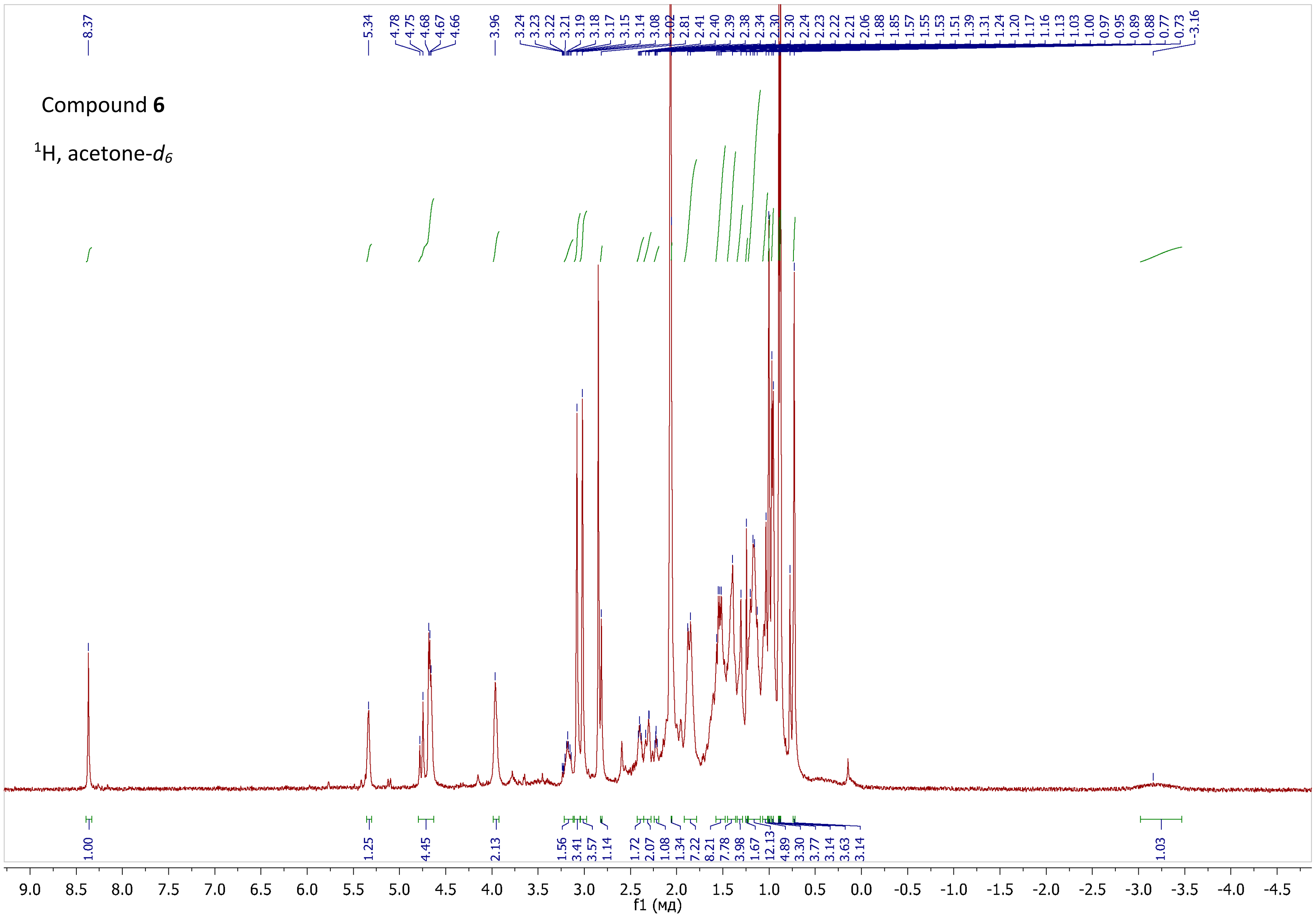

Figure S3. ^{1}H NMR spectrum of compound **6**

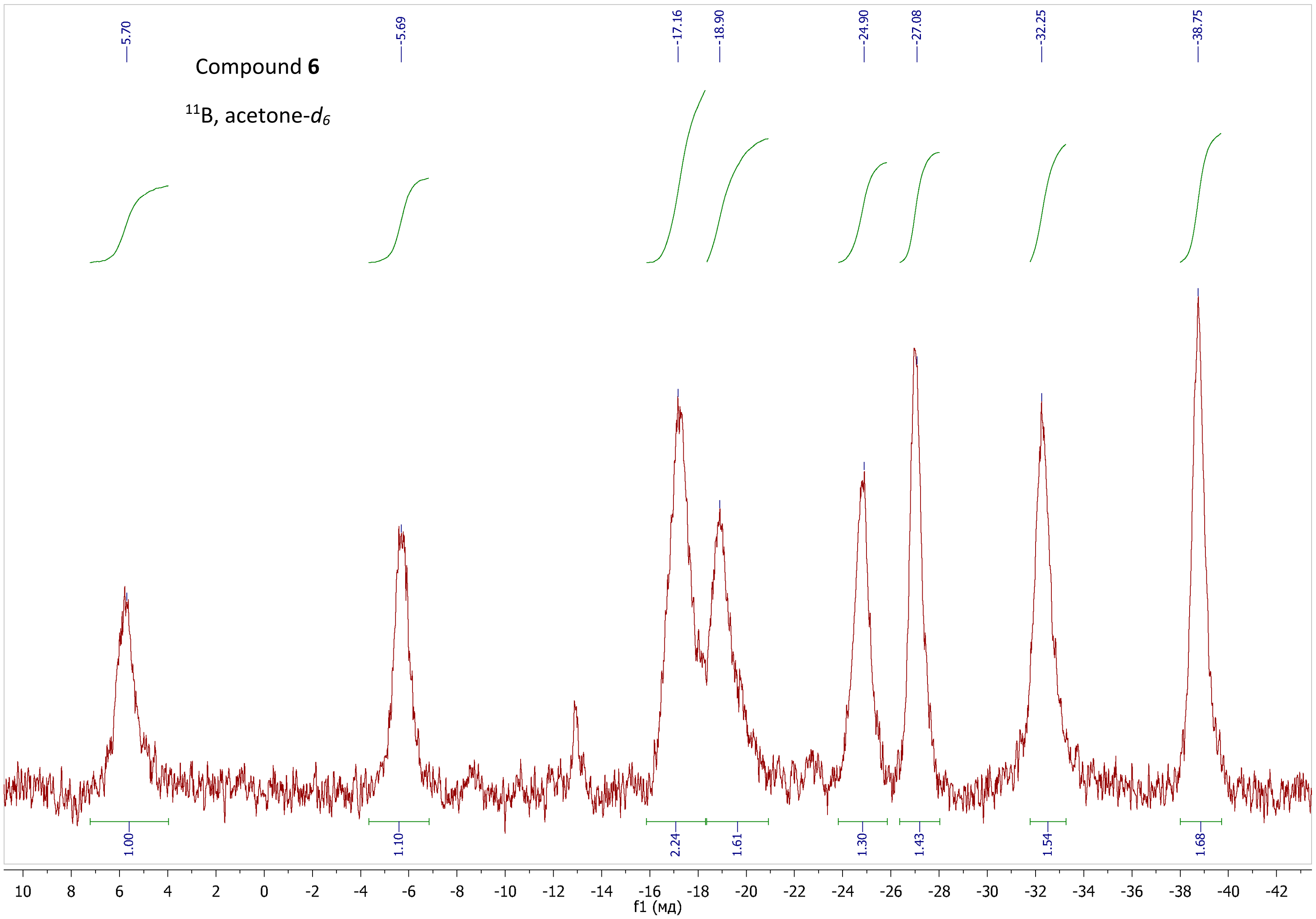

Figure S4. $^{11}B\{^{1}H\}$ NMR spectrum of compound **6**

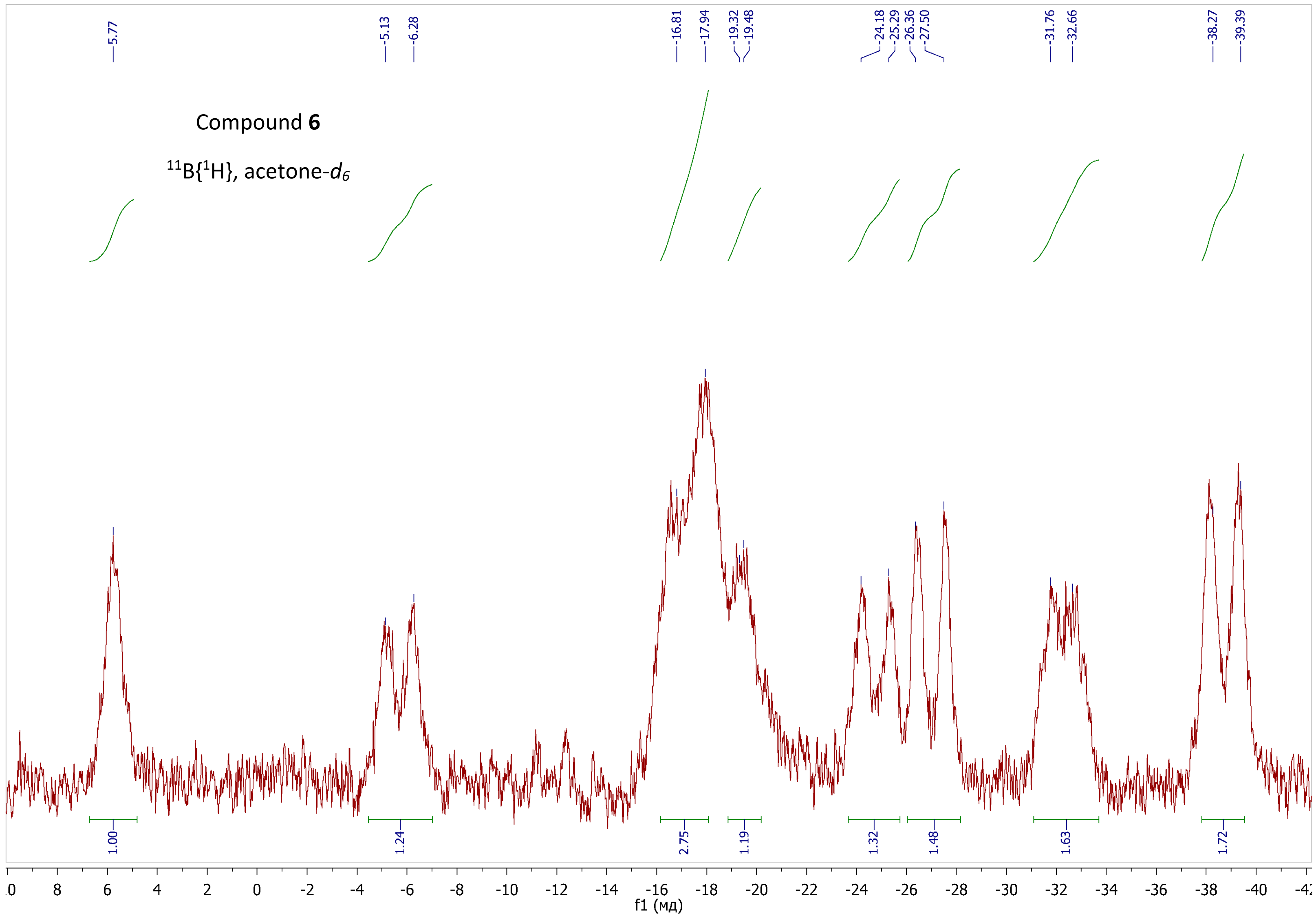

Figure S5. ^{11}B NMR spectrum of compound **6**

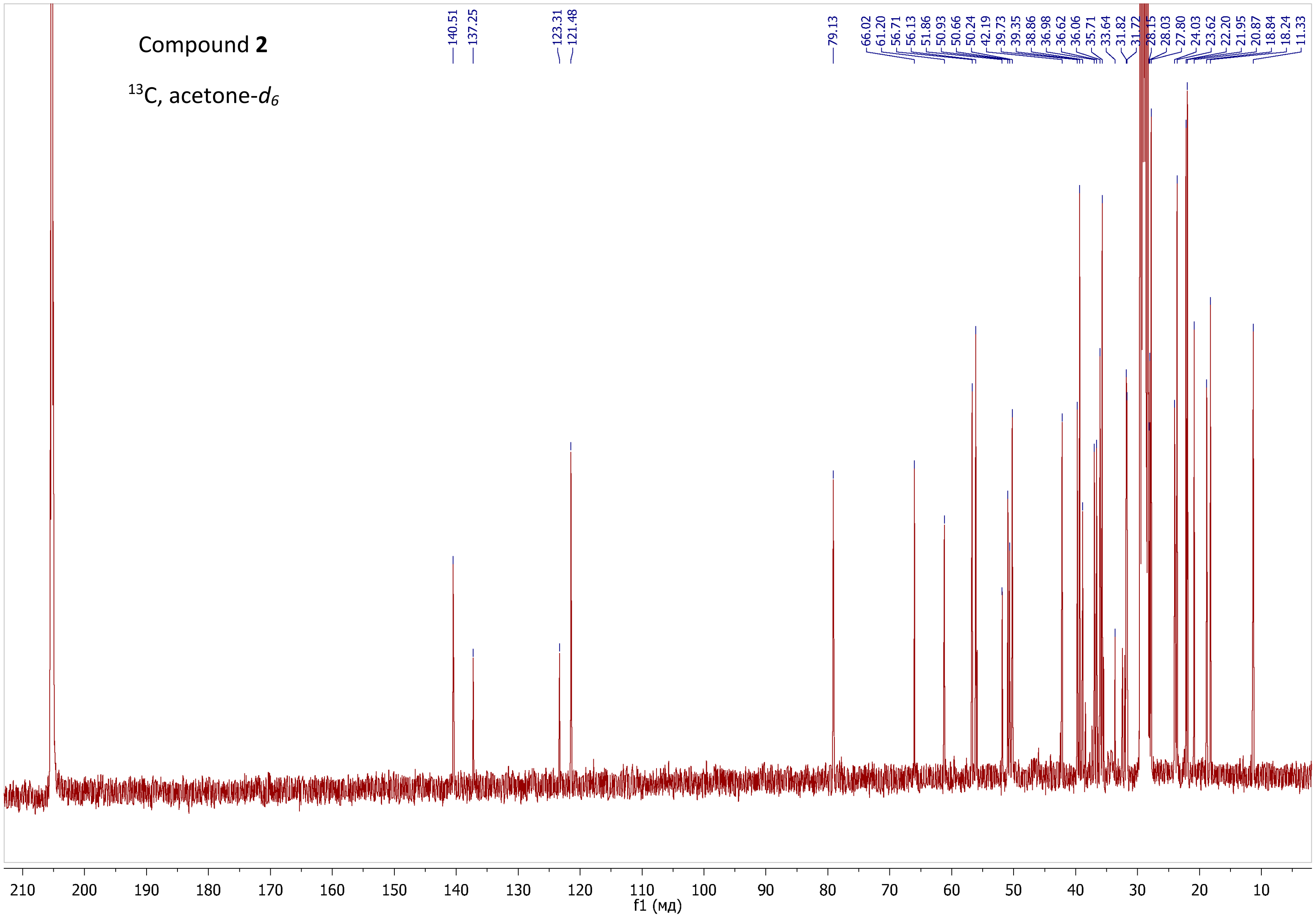

Figure S6. ^{13}C NMR spectrum of compound **6**
